# Complex post-translational regulation of the plant shikimate pathway

**DOI:** 10.3389/fpls.2026.1848534

**Published:** 2026-10-09

**Authors:** María Belén Pascual, Eloy Rivas, Vanessa Castro-Rodriguez, Ana Ginés, Jorge El-Azaz, Fernando de la Torre

**Affiliations:** 1 Departamento de Biología Molecular y Bioquímica, Universidad de Málaga, Málaga, Spain; 2 Department of Botany, University of Wisconsin - Madison, Madison, WI, United States

**Keywords:** Arabidopsis, aromatic amino acids, chloroplast, chorismate mutase, plant metabolism, post-translational modification, prephenate aminotransferase

## Abstract

Post-translational modifications (PTMs) are crucial regulators of protein function, influencing most cellular processes in both prokaryotes and eukaryotes. The fundamental effect of PTMs on metabolic control has been reported for most metabolic pathways in organisms ranging from animals and plants to bacteria and fungi. However, the impact of PTMs on the regulation of the plant shikimate pathway, which connects central carbon metabolism with the synthesis of aromatic amino acids and represents one of the largest metabolic fluxes on Earth, remains largely unexplored. In this study, we address the identification of PTMs affecting the enzymes of the shikimate pathway in *Arabidopsis thaliana*. Following a strategy based on transient expression in protoplasts, immunoprecipitation and mass spectrometric analysis, we have identified a large and varied collection of PTMs affecting the 20 alternative isoenzymes involved in the shikimate and phenylalanine (Phe) biosynthesis pathways. Our analysis revealed glycosylation, phosphorylation, methylation, nitration, and acetylation as the predominant modifications. Notably, we have identified multiple phosphorylation events that expand those previously reported. Additionally, we characterized two phosphorylation events affecting chorismate mutase 1 (AtCM1), the committed enzyme in the phenylalanine/tyrosine biosynthetic pathway. Enzymatic assays using phosphomimic recombinant pure AtCM1 support that these PTMs significantly modulate the enzyme´s allosteric behaviour, highlighting its PTMs role in fine-tuning metabolic regulation. Furthermore, functional characterization of prephenate aminotransferase (PAT) showed that phosphomimetic substitutions at Ser240 and Ser247 abolished enzymatic activity, supporting a regulatory role for phosphorylation in aromatic amino acid biosynthesis. Overall, the identification of these PTMs provides new insights into the regulation of metabolic flux through the shikimate and Phe biosynthetic pathways.

## Introduction

The aromatic amino acids (AAAs) phenylalanine (Phe), tyrosine (Tyr), and tryptophan (Trp) are required by all forms of life as essential protein components. The ability to synthesize these amino acids is restricted to plants, fungi, bacteria, and some protozoa, being absent in animals, which must obtain them through their diet. For most organisms, these amino acids also serve as precursors for the synthesis of a wide variety of specialized metabolites which, in the case of plants, include compounds of enormous interest such as the hormones indole-3-acetic acid (auxin) and salicylic acid, quinone vitamins, or very abundant structural elements like lignin. Therefore, AAA biosynthesis in plants functions as a crucial hub connecting primary and specialized metabolism.

AAA biosynthesis starts with seven linear reactions known as the shikimate pathway, where the central carbon metabolism intermediates erythrose 4-phosphate and phospho*enol*pyruvate are converted into chorismate, last common precursor to the three AAAs (**Figure 1**). Chorismate is alternatively channelled to the synthesis of Trp by the enzyme anthranilate synthase (AS), which convers chorismate into anthranilate (**Figure 1**), or to the biosynthesis of Phe and Tyr by the enzyme chorismate mutase (CM), which rearranges chorismate into prephenate (**Figure 1**). In the Phe and Tyr pathway, subsequent transamination of prephenate leads to arogenate, last common precursor for both AAAs (**Figure 1**).

**Figure 1:**
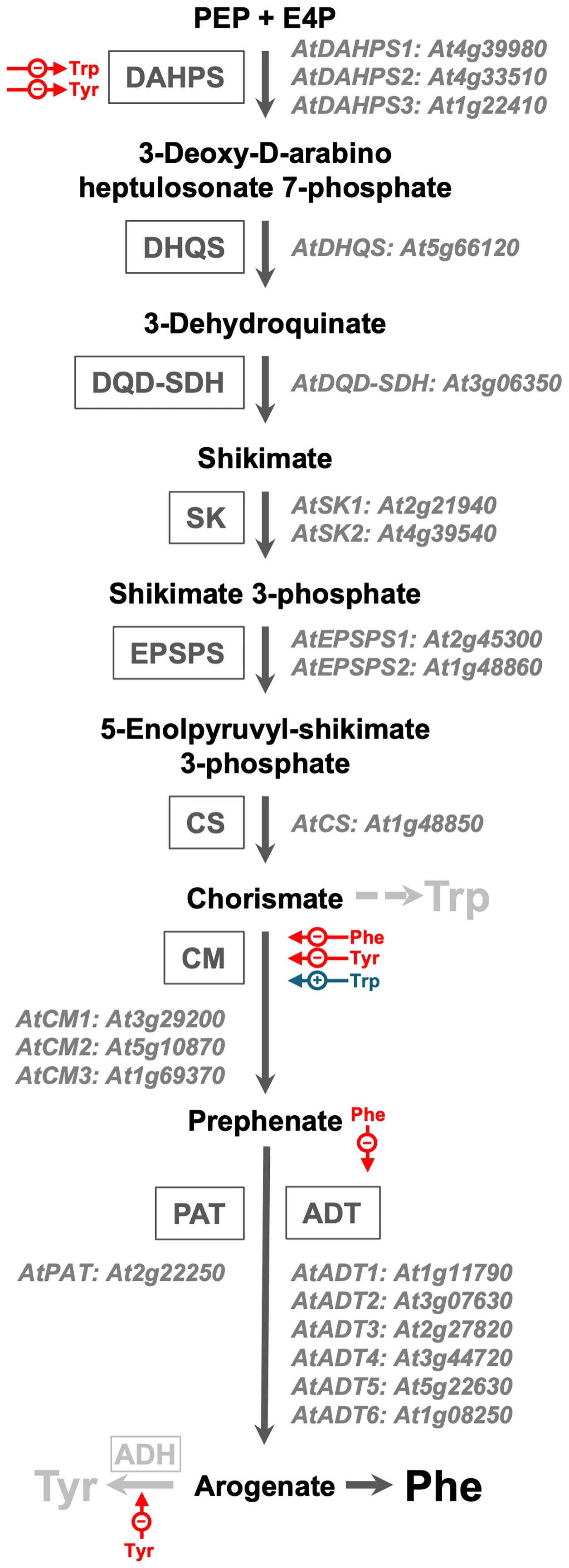
The shikimate, Phe and Tyr biosynthetic pathways pathway. Genes/loci encoding enzymes in Arabidopsis are indicated in grey. Enzymes are highlighted with white boxes. DAHPS, 3-deoxy-D-arabinoheptulosonate 7-phosphate synthase; DHQS, 3-dehydroquinate synthase; DQD-SDH, 3-dehydroquinate dehydratase–shikimate dehydrogenase; SK, shikimate kinase; EPSPS, enolpyruvylshikimate 3-phosphate synthase; CS, chorismate synthase; CM, chorismate mutase; PAT, prephenate aminotransferase; ADT, arogenate dehydratase. Red and blue arrows indicate inhibition and activation, respectively, mediated by the corresponding amino acids.

The shikimate and post-chorismate AAA pathways require a complex regulation to cope with metabolic requirements under changing physiological, developmental or environmental situations. By integrating different levels of regulation, plants are able to adapt to different temporal metabolic requirements (reviewed by El-Azaz and Maeda, 2025). Regarding transcriptional control, the transcription factor families NACs and MYBs play a central role, with NACs acting as master regulators that control the expression of MYBs, which in turn control, directly or indirectly, the expression of genes in the AAAs synthesis and downstream pathways (Mitsuda et al., 2007; Wang et al., 2011; Zhong and Ye, 2015; Pascual et al., 2018; El-Azaz et al., 2020; Orduña et al., 2022; Choung et al., 2025). Some of these transcription factors are also subjected to post-translational regulation through phosphorylation (Li et al., 2016; Gui et al., 2019; Liu et al., 2021).

The synthesis of AAAs is also subjected to a complex network of enzymatic feedback regulation by which the end products of the pathway exert control over the activities of certain enzymes. The first step of the shikimate pathway, catalyzed by the enzyme 3-deoxy-D-arabino-heptulosonate 7-phosphate synthase (DAHPS), is feedback regulated through multiple metabolites related to AAA biosynthesis (Yokoyama et al., 2021; El-Azaz et al., 2023). From chorismate, product of the shikimate pathway, alternative channelling towards Trp or Phe/Tyr pathways is controlled at the level of AS and CM (**Figure 1**), two enzymes that compete for chorismate as substrate. The activity of these enzymes is feedback inhibited by the end products of the corresponding pathways, Trp for AS and Phe and Tyr for CM. Trp is also an activator of CM; hence, Trp overaccumulation promotes metabolic flux towards Tyr and Phe biosynthesis. Similarly, enzymes catalyzing the final step of Phe and Tyr biosynthesis, arogenate deshydratase (ADT) and arogenate dehydrogenase (ADH) (**Figure 1**), are respectively feedback inhibited by Phe and Tyr. Additional regulatory mechanisms involved in the synthesis of AAAs have also been described and include subcellular compartmentalization (Eberhard et al., 1996; Widhalm et al., 2015; Qian et al., 2019; Kanaris et al., 2022; Liu et al., 2025), redox regulation (Entus et al., 2002; Tzin et al., 2012), and protease-mediated degradation by the plastidial Clp system (Nishimura and van Wijk, 2015).

Proteins can undergo multiple type of post-translational modifications (PTMs) that massively increase the number of proteoforms that can originate from a single locus. These chemical modifications can result in changes in protein function, stability, cellular and subcellular localization, or affect their interaction with other biomolecules. Multiple studies have addressed the identification of PTMs from different plant species and approaches (reviewed in Vu et al., 2018; Millar et al., 2019), which have been compiled in various databases such as Plant PTM Viewer 2.0 (Willems et al., 2024), qPTMplants (Xue et al., 2022) or PhosPhat 4.0 (Xi et al., 2021). However, most studies aimed at identifying PTMs have been non-targeted, consisting of high throughput identification of PTMs in complex protein samples. This type of approach has provided extremely valuable data, but it is not optimal to identify PTMs affecting proteins that accumulate at low levels in cells, which is the case for most enzymes involved in amino acid metabolism according to both proteomics (Heinemann et al., 2021) and transcriptomic data (Sullivan et al., 2025).

This study aimed to identify PTMs affecting enzymes in the AAA pathway in Arabidopsis, with an emphasis on phosphorylation. Owing to their low endogenous abundance, we implemented transient overexpression in Arabidopsis protoplasts, which proved to be robust and reliable. Subsequent immunoprecipitation enabled the generation of protein preparations highly enriched in the targets of interest, which allowed the identification of multiple PTMs in most enzymes by LC-MS/MS analysis (**Figure 2**). The PTMs identified here are expected to be of high interest for future studies aimed at achieving a more comprehensive understanding of the regulation of metabolic flux through aromatic compounds in plants. Notably, many of the PTMs detected here occur at evolutionarily conserved residues, suggesting potential relevance for metabolic engineering strategies. Futher, we characterized a set of selected phosphorylation events affecting the key enzymes CM and PAT which revealed that these PTMs modulate the activity and/or allosteric regulation of these enzymes, supporting the functional relevance of these PTMs in fine-tuning pathway regulation.

**Figure 2:**
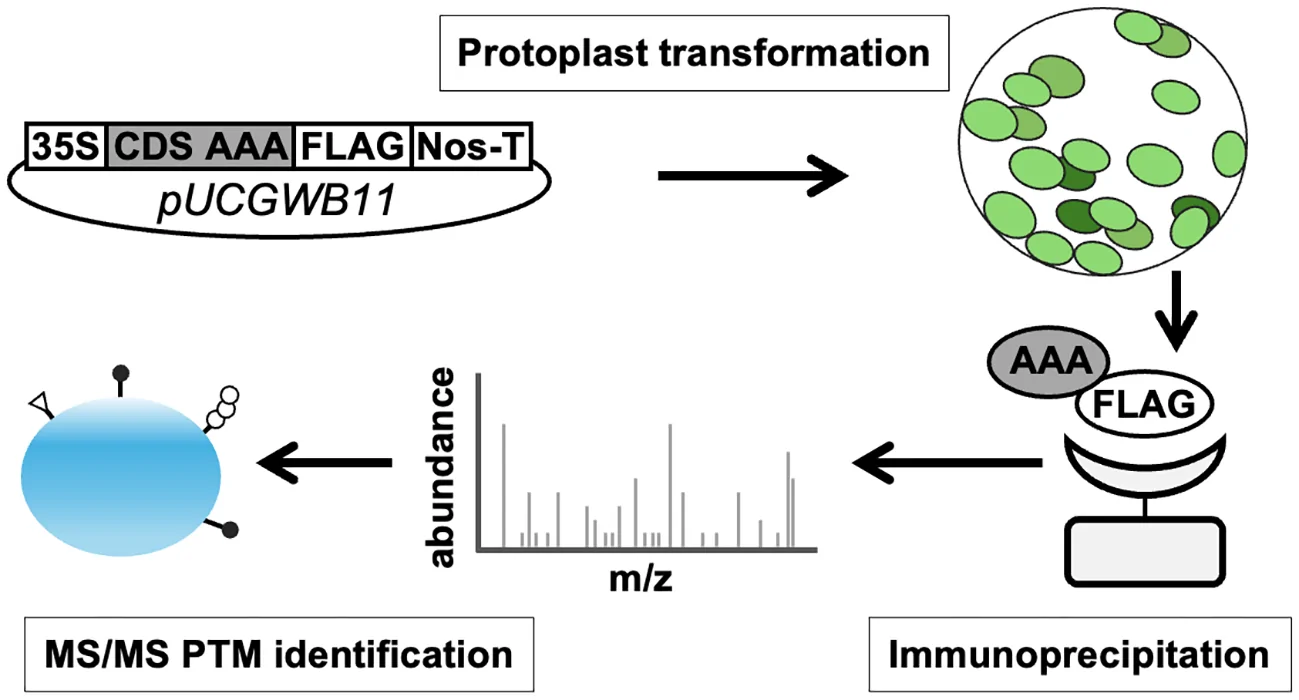
Overall work strategy. AAA pathway enzymes were produced in Arabidopsis protoplast and isolated by immunoprecipitation. PTMs were identified using LC-MS/MS.

## Results

### Transient expression in Arabidopsis protoplats allows robust overexpression of multiple AAA pathway enzymes

To generate a comprehensive dataset of PTMs affecting AAA pathway(s) enzymes, we first generated Arabidopsis transgenic plants to overexpress a total of 20 enzymatic isoforms in the shikimate and Phe/Tyr pathways of Arabidopsis (**Figure 1**). Overexpression of these enzymes was due to their naturally low levels in planta (Hildebrandt, 2018; Heinemann et al., 2021; Sullivan et al., 2025), which would otherwise prevent confident PTM identification. The genes of interest were cloned into the plant binary vector pGWB11 (Nakagawa et al., 2007) under control of the CaMV 35S promoter, and Arabidopsis plants were then transformed using the floral dipping method. Although stable overexpression lines could be successfully obtained for some enzymes, such as the three DAHPS isoforms, for several other targets, including several ADT isoforms and PAT, we were unable to obtain transgenic lines showing detectable overexpression at the transcript and/or protein level. To bypass this limitation, we decided to employ transient overexpression in Arabidopsis protoplasts as an alternative strategy to increase protein abundance and facilitate PTM identification. Firstly, overexpression of the C-terminal GFP-tagged enzymes in protoplasts confirmed that all proteins, but CM2, were targeted to the plastids (**Figure 3**), consistent with the prediction of plastid transit peptides in all of them, and previous experimental reports (Mousdale and Coggins, 1985; Rippert et al., 2009; Dal Cin et al., 2011; Maeda and Dudareva, 2012; Bross et al., 2017; Zhu et al., 2023). We next generated plasmid constructs based on the pUCGWB11 vector to overexpress, under the control of the CaMV 35S promoter, the 20 genes of interest to generate C-terminal FLAG tag fusion proteins, also in protoplasts. The use of a FLAG tag rather than GFP was deliberately chosen to facilitate the co-immunoprecipitation of potential protein interactors, as the small size of the FLAG epitope is less likely to interfere with protein–protein interactions. After PEG-mediated transformation, protoplasts were incubated under continuous light for 18 hours. The overexpressed proteins were subsequently immunoprecipitated using anti-FLAG beads. Immunoblot analysis using specific anti-FLAG antibodies confirmed that all 20 targeted proteins were successfully produced and enriched in the immunoprecipitated fractions (**Figure 4**). The resulting samples were stored for further analysis.

**Figure 3:**
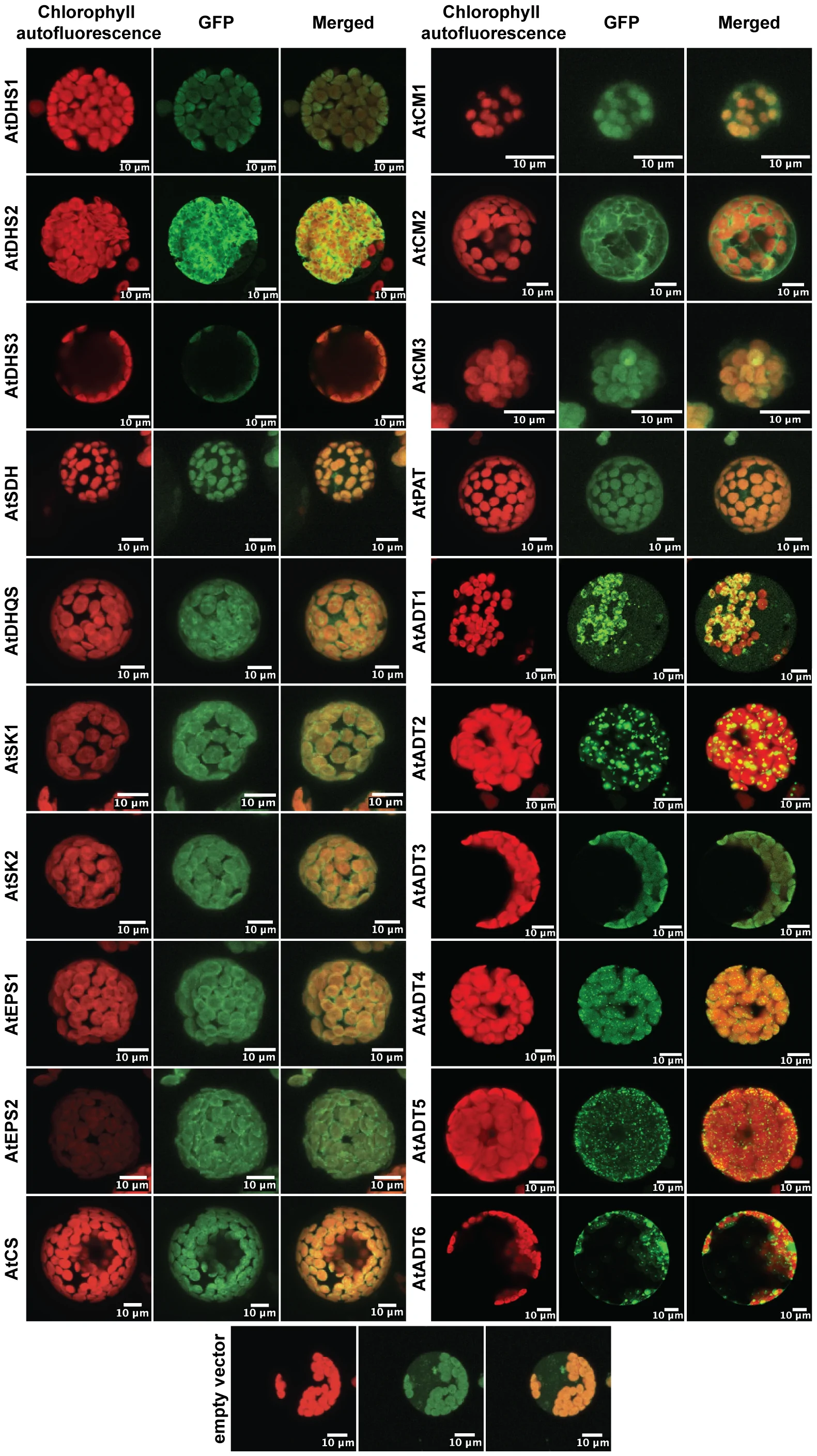
Subcellular localization of GFP-tagged proteins in Arabidopsis protoplasts. Confocal microscopy images of AAA-GFP proteins and empty vector using chlorophyll autofluorescence as an indicator of chloroplast. In each case, images of chlorophyll autofluorescence, GFP fluorescence, and merged chlorophyll and GFP fluorescence are shown.

**Figure 4:**
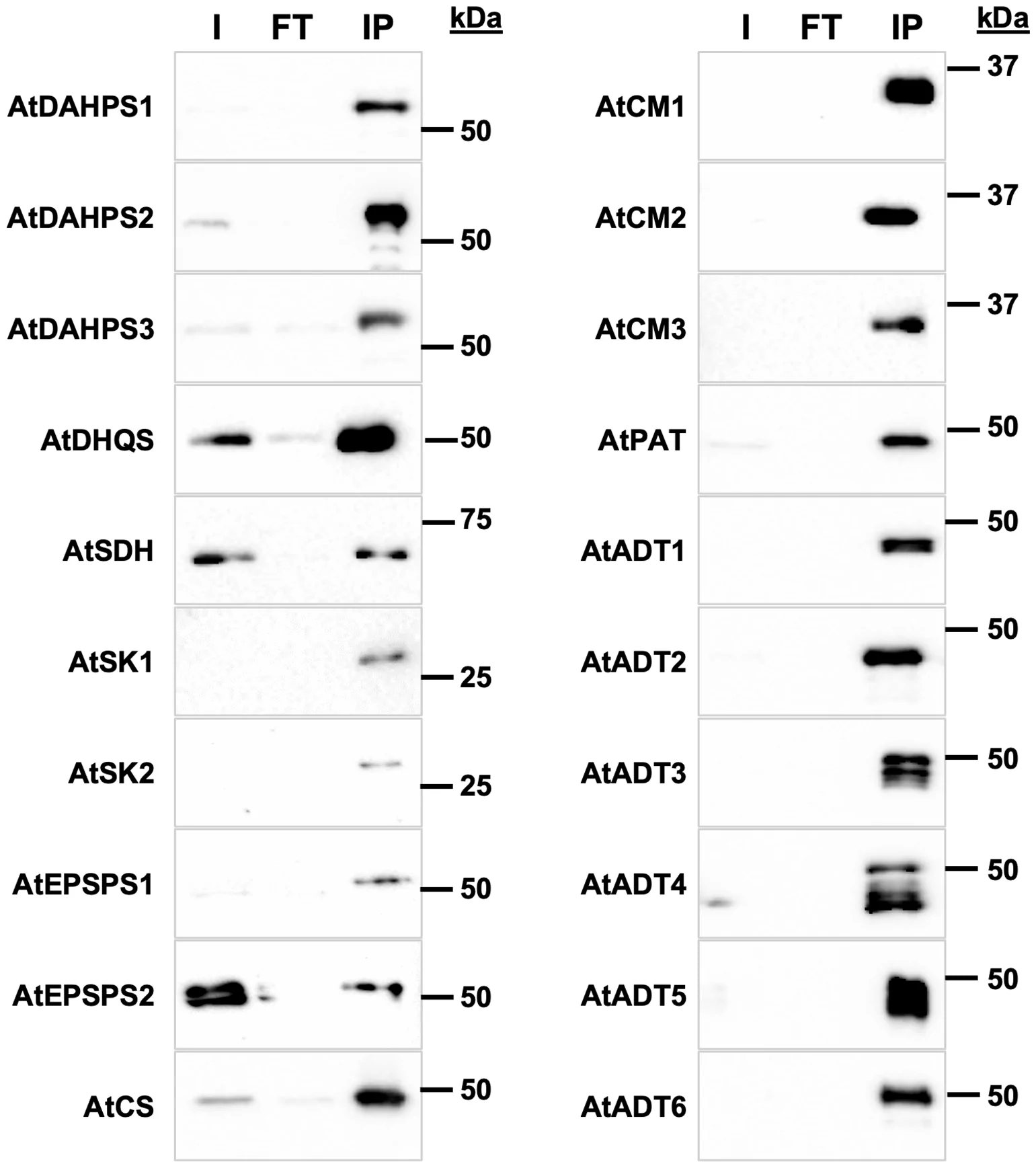
Production and immunoprecipitation of FLAG tagged proteins. Immunodetection of C-terminal FLAG tagged AAA proteins produced in Arabidopsis protoplasts and immunoprecipitated using DYKDDDDK Fab-Trap agarose beads. I, input; FT, flow through; IP, immunoprecipitated.

### LC-MS/MS uncovers a rich landscape of PTMs across AAA pathway enzymes

To proceed with the identification of PTMs affecting the proteins of interest, as well as co-purified proteins, the immunoprecipitated samples were loaded onto SDS-PAGE gels, which were run until the electrophoresis front had advanced 1 cm. Then, the gel fragment containing the whole sample was cut and sent for LC-MS/MS analysis. These analyses were designed to identify PTMs with a particular focus on phosphorylations using the PTM function implemented in PEAKS Studio (Mauser et al., 2024). In addition, this methodology enabled the detection of other PTM types with potential functional relevance to metabolic control in plant chloroplasts, particularly within AAA metabolism such as methylation, acetylation or Tyr-nitration.

LC-MS/MS data showed that the most frequently identified PTM was the methylation of Lys and Arg residues, which was detected in all AAA enzymes but AtADT1 and AtSK2 (**Table 1**). These PTMs, beyond their epigenetic roles (Kouzarides, 2002; Sims et al., 2003; Liu et al., 2010; Nakamura et al., 2025), also take place in non-histone proteins across different taxa and have been reported in plant plastid proteins (Alban et al., 2014; Lehtimäki et al., 2015). Together, the total number of methylations detected was 149, with 75 corresponding to single Lys methylations, 61 to single Arg methylations, 8 to Lys dimethylations, and 5 to Arg dimethylations. Except for two sites in AtADT6, Lys and Arg methylations occurred in the mature proteins rather than in the predicted chloroplast transit peptides (cTP). AtDHQS harbored the largest number of methylation events, with a total of 18 sites identified.

**Table 1:** PTMs detected in the enzymes of the Shikimate and Phe-synthesis pathways.

Protein	AGI code	Phosphorylation	Lys acetylation	N-terminal acetylation	K/R methylation	Tyrosine nitration	Ubiquitination
DAHPS1	At4g39980	Y228, T229	K144, K360, K382, K400, K428	A2	Monomethyl: K144, K194, K203, K215, R217, R223, R351, R385, K428, R446 Dimethyl: K203	Y228, Y246, Y483	K360
DAHPS2	At4g33510	Y223	K341, K359	–	Monomethyl: K121, K192, R228, R315, R328, R362	Y205, Y223, Y460	–
DAHPS3	At1g22410	Y246, T416, T426, T492	K144, K185, K360, K400, K428	M1	Monomethyl: K64, K72, K144, R187, K194, R251, R262, R338, R351, R385, K428	–	–
DHQS	At5g66120	S62, S77, S310	K153, K299, K305, K314, K370, K395	M1	Monomethyl: R73, R88, K129, K153, K155, K162, R199, R227, K266, K281, R300, K305, K314, R319, K370, R371, K380, K415, R429 Dimethyl: K305, K415	–	–
DHDSDH	At3g06350	S94, S104, Y326	K107, K208, K331, K454, K473, K478	–	Monomethyl: K107, R126, K140, R170, K208, R231, K241, R254, R274, K400, R577	–	–
SK1	At2g21940	–	–	M1	Monomethyl: K93, K98, R246	–	–
SK2	At4g39540	–	–	–	–	–	–
EPSPS1	At2g45300	T383	K466	–	Monomethyl: R149, R235, K368, K372, K466, K469, R480 Dimethyl: K368	–	–
EPSPS2	At1g48860	S166	K467	M58	Monomethyl: R150, R236, K369, K373, K468, K470 Dimethyl: K369, R481	–	–
CS	At1g48850	S148, S154, S258, S292	K163, K288, K354	–	Monomethyl: R82, R96, R152, K163, R268, K288, R350, R388 Dimethyl: R82, K163	–	–
CM1	At3g29200	S184, T215, S227, S243, Y308	K143, K228, K253, K295, K309	M1	Monomethyl: K78, R79, K206, K228, R229, K234, K257, R282, K289, K295, K309 Dimethyl: K228, R229, K309	–	–
CM2	At5g10870	S11, T72, S223	K78, K112, K165, K171, K199, K211, K220, K230, K234	V4	Monomethyl: R23, K42, R54, R81, K104, R184, K211, K220, K230	–	K165, K171
CM3	At1g69370	–	K126, K223	–	Monomethyl: K188, K236 Dimethyl: K188	–	–
PAT	At2g22250	S240, S242, S247	K228, K330, K345, K366	A2	Monomethyl: R142, K150, K213, K223, K251, K322, K330, K345, K355, K366, R369, R445, K461	Y132, Y249, Y317, Y448	K228, K345, K377, K383
ADT1	AT1G11790	–	K229, K243	–	–	–	–
ADT2	AT3G07630	–	K213	–	Monomethyl: K82, K117, K213, K246, R269	Y110, Y161, Y251	K213, K249
ADT3	AT2G27820	S119, Y125, S221, T232, S344	K235	–	Monomethyl: K52, R122, K139, R181, K235, R261, K320 Dimethyl: R122	–	–
ADT4	AT3G44720	S53, S178, T316	K65	M1	Monomethyl: K65, K78, R126, K143, R185, R246, K326, R404	–	–
ADT5	AT5G22630	S61, T234, S237	K240	–	Monomethyl: K69, K144 Dimethyl: K240	–	–
ADT6	AT1G08250	S8, S29, S173, T357	K41, K134	–	Monomethyl: R25, R28, K41, K83, R94, K134, K230, R256, K315, R393	–	–

Lys acetylation was detected in all AAA pathway proteins, except the two isoforms of shikimate kinase (SK; **Figure 1**). This PTM has been linked to fundamental aspects of metabolic control in plants, including the Calvin cycle (Finkemeier et al., 2011; Gao et al., 2016), photosynthesis light reactions (Hartl et al., 2017) or pyruvate dehydrogenase (Neusius et al., 2022). The total number of Lys acetylations was 56 with cytosolic AtCM2 having the highest number of acetylations at nine (**Table 1**). We also detected N-terminal acetylation in 9 out of 20 of the proteins analyzed. With the sole exception of AtEPSPS2, this PTM was always found in the N-terminus of the cTP (**Table 1**).

Tyrosine nitration, a PTM that regulates multiple plant enzymes (Lozano-Juste et al., 2011; Begara-Morales et al., 2013; Sainz et al., 2015; Kolbert et al., 2017) was detected in four enzymes: AtDAHPS1, AtDAHPS2, AtADT2 and AtPAT. Interestingly, AtDAHPS1 and AtDAHPS2 shared the same three nitrosylated Tyr residues. Further, we also identified Lys ubiquitination sites in several AAA enzymes, specifically one in AtDAHPS1, (Lys360), two closely located residues in AtCM2, (Lys165 and Lys171), four in AtPAT (Lys228, Lys345, Lys377 and Lys383) and two in AtADT2 (Lys213 and Lys249) (**Table 1**).

Lastly, we identified a total of 45 phosphosites (the main focus of this study) in 15 out of the 20 proteins analyzed. Among these phosphosites, 28 corresponded to Ser residues, 11 to Thr and six to Tyr (**Table 1**). Quite remarkably, the vast majority of all these phosphorylations, 43 out of 45, had not been previously described. No phosphosites were found in the proteins AtSK1, AtSK2, AtCM3, AtADT1 and AtADT2, possibly due to lower total peptide abundance for these enzymes, which may be related to lower expression in protoplasts or a less efficient immunoprecipitation compared to other proteins. In contrast, we identified the highest number of phosphorylations in the enzymes AtADT3 and AtCM1, having five each. All these PTMs were localized within the mature region of the proteins, except for Ser8 and Ser29 from AtADT6, which were located within the predicted cTP.

### Ser223Asp phosphomimic version of AtCM2 shows altered cytosolic distribution in Arabidopsis protoplats

We next focused our investigations in the PTMs identified in AtCM1 and AtCM2, as CM is a highly regulated committed enzyme that modulates metabolic flux into the Phe/Tyr pathway (**Figure 1**, El-Azaz and Maeda, 2025). Regarding AtCM1, we selected for further analysis two phosphorylation events detected in AtCM1, Ser184 and Ser244, to evaluate their potential contribution to the regulation of the activity of this enzyme. These residues were selected based on their evolutionary conservation across plant CMs (**Supplementary Figure 1**), and their position in the 3D structure of the protein (**Figure 5**). Based on the 3D structure of AtCM1 (Westfall et al., 2014), both residues are solvent exposed on the surface of the AtCM1 protein, and therefore accessible for modification (**Figure 5A**). We determined that Ser244 is located in a narrow region connecting α-helix 5 and α-helix 4, which houses various residues involved in the allosteric regulation of the enzyme (**Figure 5B**). We also observed that Ser184 is part of a short α-helix located close to a section of α-helix 8 containing critical residues in the active site of the enzyme (Westfall et al., 2014) (**Figure 5C**). Relative to AtCM2, we selected two phosphosites detected in this study, Thr72 and Ser223. According to a homology-based structural model for CM2, Thr72 is a highly conserved amino acid residue in plant CMs and is located in the α-helix 2, which forms part of the contact surface between the two monomers of the enzyme (**Figure 6**). In contrast, Ser223 of AtCM2 is included within a hypervariable region of plant CMs (**Supplementary Figure 1**) found between α-helices 7 and 8, at the protein dimerization surface. Based on these models, we hypothesized that the phosphorylation status of these residues could impact the functionality of CM, in particular by affecting the kinetic properties of AtCM1, and the oligomerization state of AtCM2.

**Figure 5:**
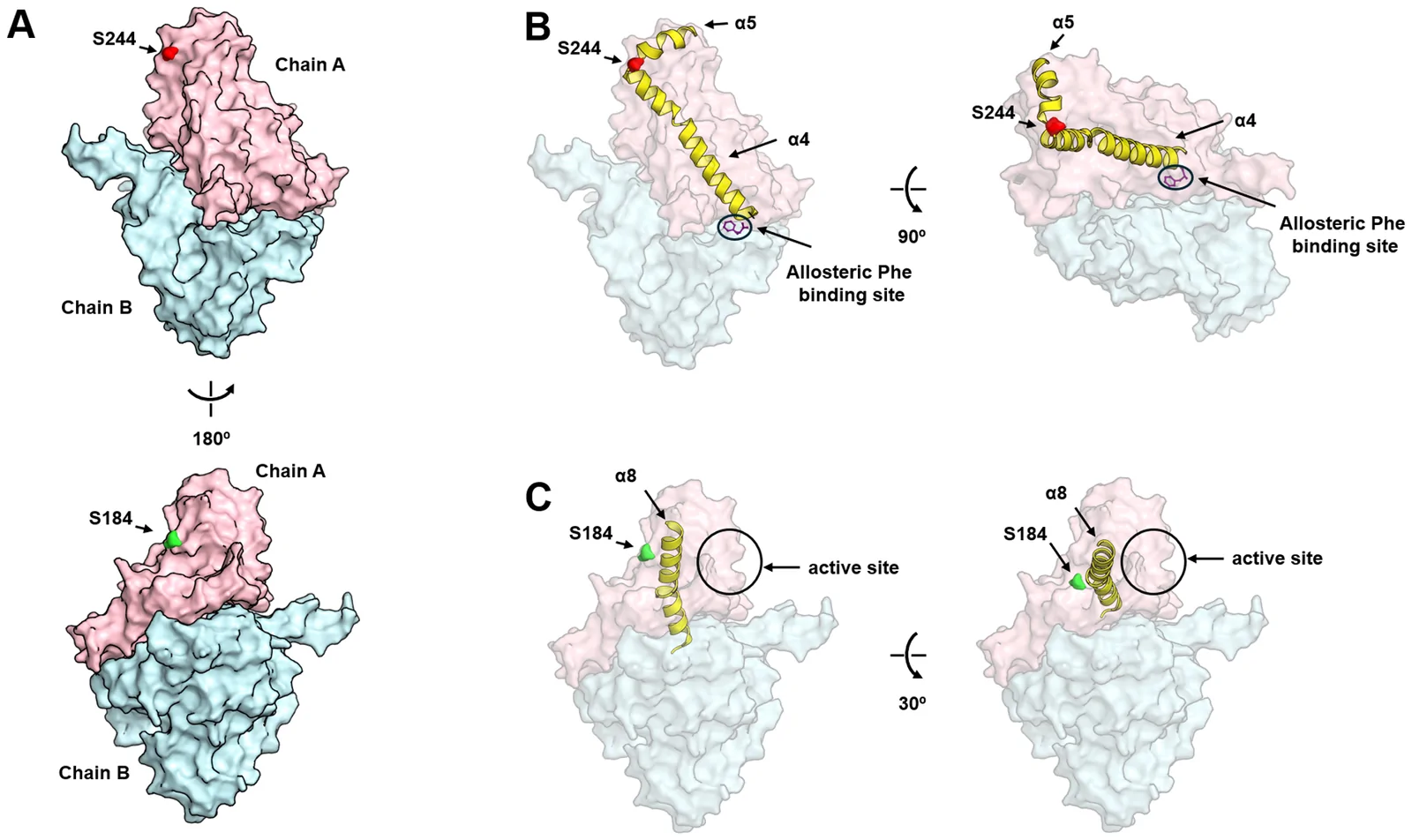
Location of the phosphosites Ser184 and Ser244 in the 3D structure of AtCM1. **(A)** surface structure of the AtCM1 homodimer formed by chains **(A, B)** In red location of Ser244 (upper panel) and in green location of Ser184 (lower panel). **(B)** Detailed location of Ser244 relative to helix 4 and 5 and allosteric Phe binding site. **(C)** Detailed location of Ser184 relative to helix 8 and active site. The images, corresponding to PDB file 4PPV, have been rendered using PyMOL 3.1.

**Figure 6:**
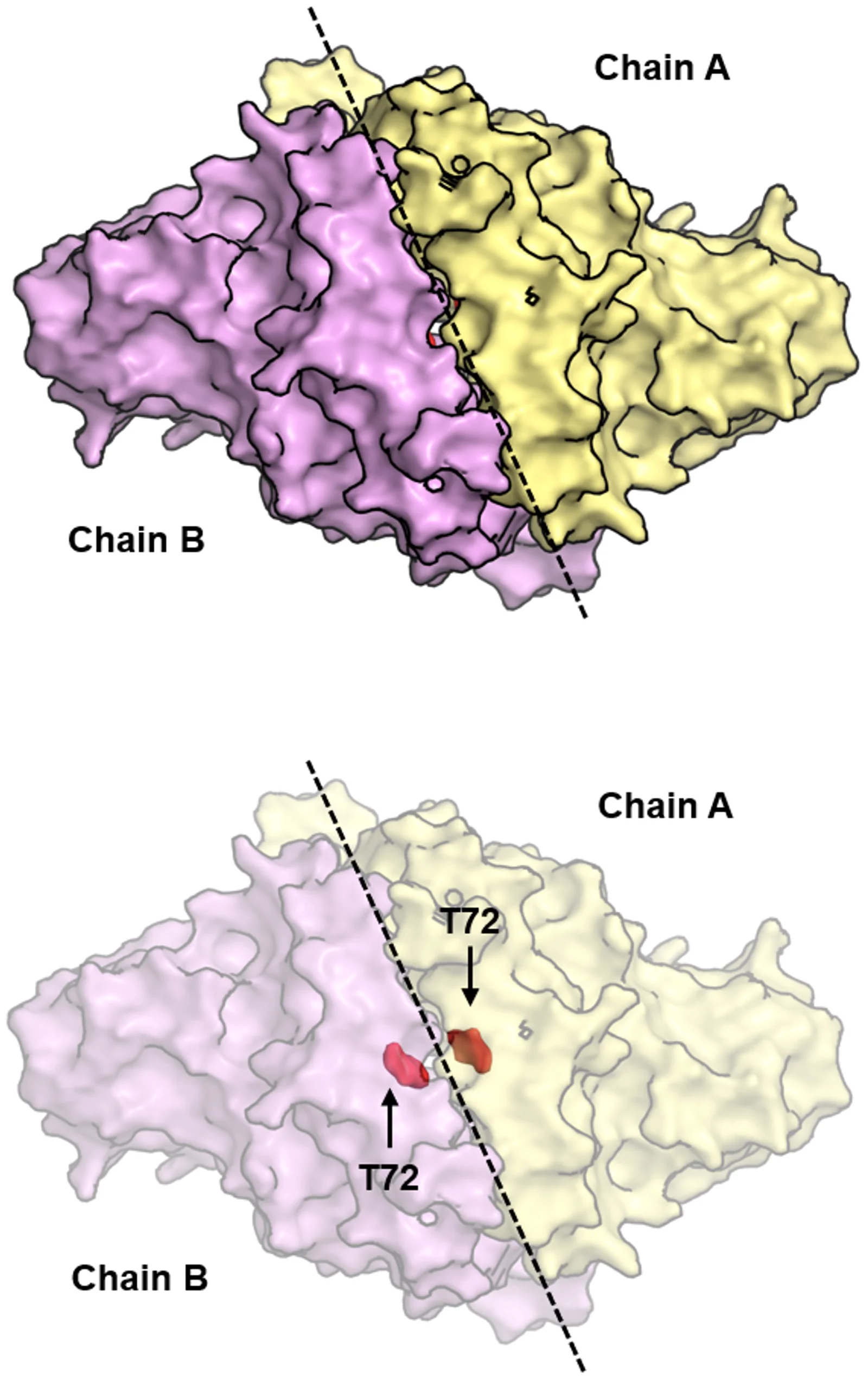
Location of the phosphosite Thr72 in the 3D structure of AtCM2. Upper panel, surface structure of the AtCM2 homodimer formed by chains (A, B) Lower panel, location of Thr72 relative to homodimerization surface. The images, corresponding to PDB file 4PPV, have been rendered using PyMOL 3.1.

To investigate whether these phosphoresidues could impact the *in vivo* function of AtCM1 and AtCM2, and in particular their subcellular localization, we generated phosphomimetic variants of AtCM1 and AtCM2 by substituting Ser or Thr with Asp at positions Ser184 and Ser244 in AtCM1, and Thr72 and Ser223 in AtCM2. These phosphomimetic variants were then overexpressed in Arabidosis protoplasts as C-terminal GFP fusion proteins, side-by-side with their corresponding wild type controls. Confocal laser scanning microscopy imaging revealed that AtCM1 phosphomimic mutations Ser184Asp and Ser244Asp showed no discernible impact on GFP signal accumulation compared to wild type AtCM1, with all fusion proteins showing clear plastid localization (**Figure 7**). Regarding AtCM2, the observed cytosolic accumulation pattern remained unaltered for the AtCM2 enzyme carrying the Ser223Asp point mutation (**Figure 7**). Conversely, AtCM2^T72D^ showed a characteristic pattern consisting of small cytoplasmic puncta (**Figure 7**), suggesting that Thr72Asp mutation may alter the oligomerization state of CM2 or its interaction with other proteins.

**Figure 7:**
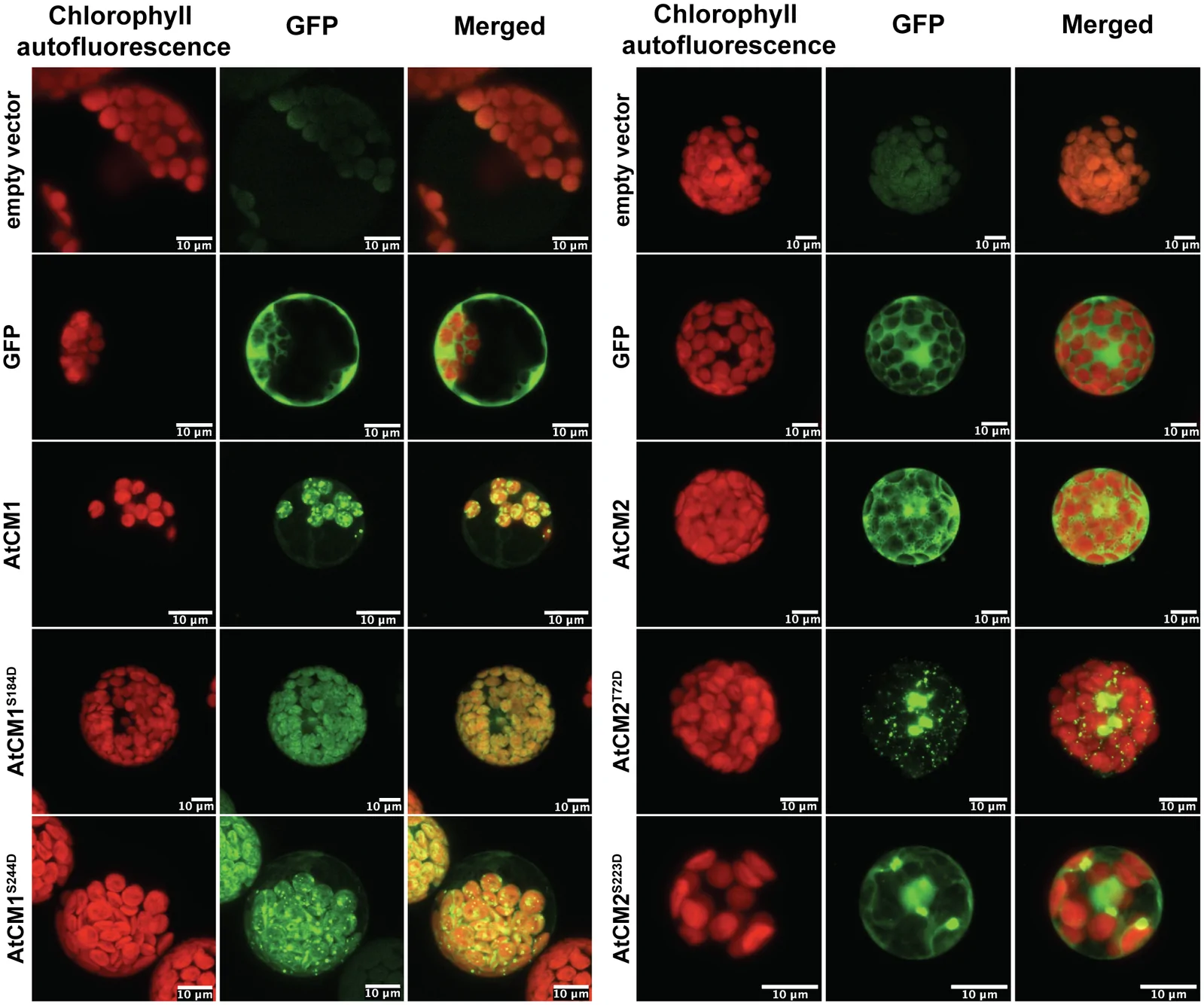
Confocal GFP imaging of proteins AtCM1, AtCM2 and phosphomimetic variants in Arabidopsis protoplasts. Confocal microscopy images of CM proteins and empty vector using chlorophyll autofluorescence as an indicator of chloroplast. In each case, images of chlorophyll autofluorescence, GFP fluorescence, and merged chlorophyll and GFP fluorescence are shown.

### Phosphorylation of residues Ser184 and Ser244 fine-tunes the activity of plastidial AtCM1

To investigate if Ser184 and Ser244 phosphorylations may have an impact on the activity and allosteric regulation of AtCM1, as suggested by our *in silico* modelling (**Figure 5**), we cloned the wild-type and phosphomimetic forms Ser184Asp and Ser244Asp of AtCM1, without the predicted cTP, into the *E. coli* expression vector pDEST17, and purified them in recombinant form using the N-terminal 6xHis tag. We first determined the kinetic parameters of the wild-type recombinant enzyme (i.e., *K*_m_ and *k*_cat_) by measuring its activity at varied concentrations of chorismate between 0 and 5 mM. For the wild-type AtCM1, the *K*_m_ for chorismate was 0.6 mM, and the *k*_cat_ was 200.8 s^-1^, resulting in a catalytic efficiency (*k*_cat_/*K*_m_) of 334,667 M^-1^s^-1^ (**Table 2**; **Supplementary Figure 2**). Consistent with Ser184Asp being located near the active site (**Figure 5**), AtCM1^S184D^ showed a significantly reduced *k_cat_* of 96.1 s^-1^ whereas its *K*_m_ remained unchanged with a value of 0.55 mM together resulting in a reduction of the catalytic efficiency *k*_cat_/*K*_m_ to a value of 174,727 M^-1^s^-1^ (**Table 2**; **Supplementary Figure 2**). In the opposite direction, AtCM1^S244D^ showed a marked increase in its *k_cat_* to 392.5 s^-1^ whereas its *K_m_*, 0.7 mM, was mainly unaltered, resulting in an increased catalytic efficiency of 560,714 M^-1^s^-1^ (**Table 2**; **Supplementary Figure 2**).

**Table 2:** Kinetic parameters of AtCM1 and mutant versions S184D and S244D.

Protein	*k_cat_* (s^-1^)	*K_m_* (mM)	*K_cat_/K_m_* (M^-1^ s^-1^)
AtCM1	200.8 ± 28.2	0.6 ± 0.2	334,667
AtCM1^S184D^	96.1 ± 10.5	0.55 ± 0.2	174,727
AtCM1^S244D^	392.5 ± 22.8	0.7 ± 0.1	560,714

We then evaluated the putative effect of the Ser184Asp and Ser244Asp phosphomimetic mutations on the AAA-mediated feedback regulation of AtCM1. To this end, we carried out activity measurements of these proteins in the presence of 2 mM of Phe, Tyr or Trp, or no added AAAs as control (**Figure 8**). Our results confirmed previous reports describing a marked inhibition mediated by Phe and Tyr (Westfall et al., 2014). Whereas AtCM1^S184D^ was statistically equal to wild-type AtCM1, AtCM1^S244D^ showed enhanced sensitivity to inhibition by both Phe and Tyr (**Figure 8**). Thus, 2 mM Phe inhibited wild-type AtCM1 by ~29%, but inhibited AtCM1^S244D^ to ~51%. Similarly, 2 mM Tyr inhibited wild-type AtCM1 by ~43%, compared to ~69% inhibition in AtCM1^S244D^. We did not observe, however, significant changes in sensivity to Trp (**Figure 8**). Changes in the allosteric regulation of AtCM1^S244D^ would be consistent with Ser244 being located close to the allosteric regulatory binding site of the enzyme (**Figure 5B**).

**Figure 8:**
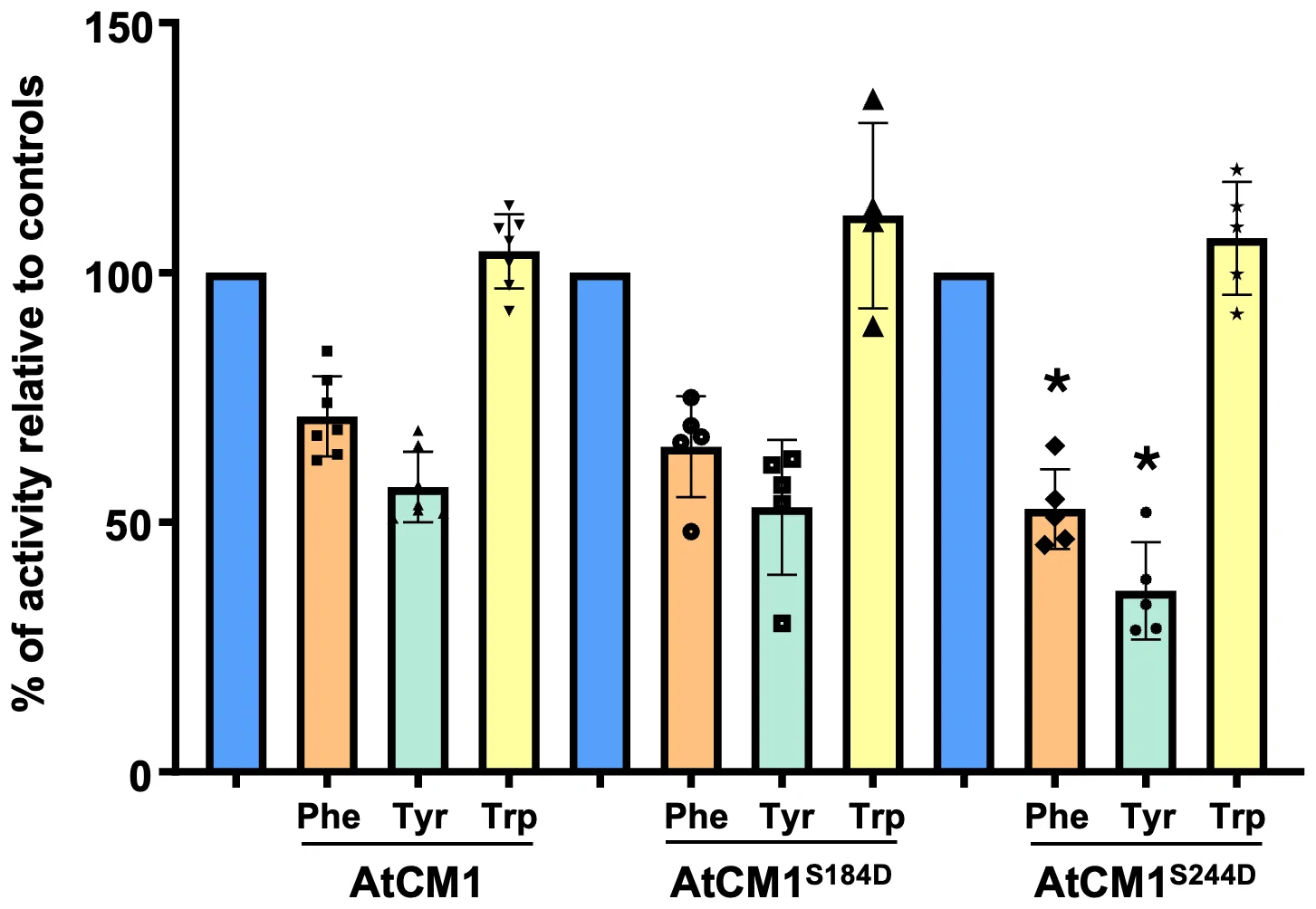
Effect of amino acids on the activity of AtCM1 and phosphomimetic versions. All values are expressed as a percentage of the no effector activity of each protein. Concentrations for assays were 0.5 mM chorismate and 2 mM of the corresponding effector. Bars indicate average activity, expressed as % of the water control, calculated from n = 5–7 independent assays. Error bars correspond to standard deviation. Asterisks indicate significant differences compared to wild-type protein according to Student’s t-test (P < 0.05).

### Phosphomimetic substitutions at Ser240 and Ser247 abolish PAT activity

Additionally, we analyzed the effect of two phosphorylation PTMs found in the enzyme PAT, which catalyzes the last common step in Phe and Tyr biosynthesis (**Figure 1**). Among the identified PTMs (**Table 1**), phosphorylation was detected at Ser240 and Ser247. Both residues are located within a highly conserved region across plant PATs and, based on available crystal structures in the open conformation (PDB: 6F5V; Giustini et al., 2019), map to an internal region adjacent to the protein surface (**Figure 9**). To evaluate the functional significance of these PTMs, we expressed and purified recombinant phosphomimetic PAT mutants alongside the wild-type (WT) enzyme in *Escherichia coli*. In all cases, the recombinant proteins were produced without the first 60 amino acids at the N-terminus, which correspond to the predicted plastid transit peptide. Enzyme assays under saturating conditions of substrates, ran side-by-side using wild type PAT enzyme as control, revealed that both phosphomimetic mutants exhibited a complete loss detectable PAT activity (**Figure 9**). We also analyzed the subcellular localization of the PAT phosphomimetic variants by transiently expressing the corresponding C-terminal GFP fusion proteins in protoplasts, alongside the wild-type PAT protein as a control. Confocal laser scanning microscopy revealed no discernible differences in GFP signal distribution between the phosphomimetic variants and the wild-type protein, with all proteins showing the expected plastid localization (**Supplementary Figure 3**).

**Figure 9:**
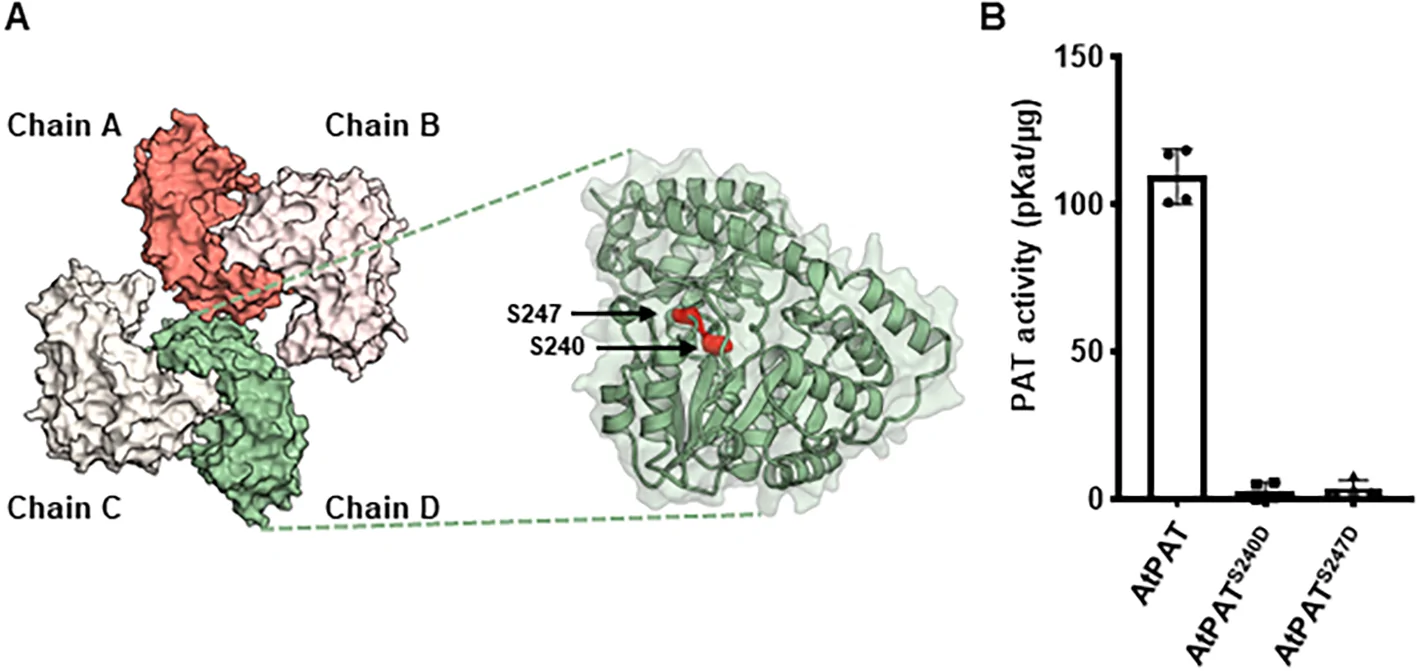
Structural and functional characterization of Ser240 and Ser247 phosphosites in AtPAT. **(A)** Location of the phosphosites Ser240 and Ser247 in the 3D structure of AtPAT. Left panel, surface structure of the AtPAT homotetramer formed by chains (A–D) Right panel, location of Ser240 and Ser247 in the monomer (D) The images, corresponding to PDB file 6F5V, have been rendered using PyMOL 3.1. **(B)** Determination of PAT activity in recombinant wild-type and phosphomimic mutant proteins AtPATS240D and AtPATS247D. Error bars correspond to standard deviation.

## Discussion

Plants require a precise control of AAA biosynthesis to cope with the dynamically changing requirements for downstream metabolites, such as phenylpropanoids, hormones, and others. Extensive evidence indicates that AAA synthesis is subjected to tight regulation at the transcriptional level and also by the concentrations of its end metabolites; however, no evidence has yet demonstrated regulatory mechanisms based on chemical modifications of the enzymes. Notably, PTMs like phosphorylation events have been reported as important regulatory elements in enzymes that are closely connected to AAA metabolism such as tryptophan aminotransferase (TAA) and phenylalanine ammonia lyase (PAL), but also in transcription factors involved in controlling the metabolism of AAAs including LTF1, MYB75 and NST1 (Li et al., 2016; Fantino et al., 2017; Gui et al., 2019; Wang et al., 2020; Liu et al., 2021).

Despite the existence of numerous published works focused on the high-throughput identification of PTMs in plants (reviewed in Vu et al., 2018; Millar et al., 2019), and databases where these modifications are compiled (Xi et al., 2021; Xue et al., 2022; Willems et al., 2024) these large-scale studies have been poorly effective in identifying PTMs in AAA enzymes, probably due to their low levels in plants. To overcome this limitation, we initially attempted to generate stable Arabidopsis overexpression lines for the enzymes analyzed in this study. Although stable overexpression lines were successfully obtained for some targets, for several others, including multiple ADT isoforms and PAT, we were unable to recover lines showing detectable overexpression at the transcript and/or protein level. The reasons underlying the difficulty in obtaining these lines remain unclear but may potentially result from detrimental metabolic perturbations. Therefore, we opted to transiently overexpress the proteins in Arabidopsis protoplasts. This strategy enabled the production of substantial amounts of the target proteins, which, after immunoprecipitation and LC-MS/MS analysis, lead us to the identification of multiple PTMs in all 20 studied Arabidopsis proteins except AtSK2, likely due to its low expression in protoplasts. As we have shown in **Figure 3**, all enzymes but AtCM2 are located in chloroplasts, however, we cannot rule out that some PTMs originate from proteins en route to chloroplasts; notably, ubiquitination was detected in several enzymes, a modification rarely associated with plastids (van Wijk and Adam, 2024).

Here, we focused our attention on types of PTMs considered to be of greater importance in chloroplasts: Ser/Thr/Tyr phosphorylation, Lys acetylation, N-terminal acetylation, Lys/Arg methylation, Tyr nitration. With regard to phosphorylations, we identified 45 phosphoresidues distributed across 15 of the 20 enzymes in the pathway. Although there was no evidence to date supporting the regulation of AAA pathway enzymes by this PTM, phosphorylation has a fundamental role in the regulation of other pathways in the plastid stroma, such as the Calvin-Benson-Basham cycle and starch metabolism (reviewed in Grabsztunowicz et al., 2017). Importantly, several studies have suggested that a relatively small number of protein kinases and phosphatases are responsible for the majority of chloroplast (de)-phosphorylation events (Schliebner et al., 2008; Bayer et al., 2012; Richter et al., 2016). This relevant aspect may facilitate the future identification of the mechanisms underlying the phosphorylation-dependent regulation of AAA-pathway enzymes.

We detected extensive Lys acetylation, a modification that in chloroplasts occurs via enzymatic and non-enzymatic mechanisms and regulates key enzymes such as RuBisCO and pyruvate dehydrogenase (Gao et al., 2016; Bienvenut et al., 2020; Neusius et al., 2022). Interestingly, Lys acetylation was detected at 6 of the 9 ubiquitinated residues identified, suggesting a regulatory mechanism that may control alternative degradation or stabilization of AAA proteins. We have also detected that 8 of the AAA enzymes are N-acetylated at the N-terminal chloroplast transit peptide, corresponding to their cytosolic forms. Studies report both stabilizing (Bienvenut et al., 2011; Möllenbeck et al., 2025) and destabilizing (Eckardt et al., 2024) effects of N-terminal acetylation on protein half-life, suggesting that this PTM may underlie an unexplored mechanism of proteostatic control in the AAA pathway. A putative regulatory role may be also postulated for the most abundant PTM detected in this work, Arg and Lys methylation, a modification widely described in plastids affecting different metabolic pathways (Alban et al., 2014; Lehtimäki et al., 2015). Additionally, Tyr nitration, detected here in four enzymes (AtDAHPS1, AtDAHPS2, AtPAT, and AtADT2), may modulate enzyme activity (Lozano-Juste et al., 2011; Begara-Morales et al., 2013; Sainz et al., 2015). Notably, AtDAHPS1 and AtDAHPS2 exhibited similar nitration patterns, and in both proteins one of the nitrated residues (Tyr228 in AtDAHPS1 and Tyr223 in AtDAHPS2) was also identified as a phosphorylation site, suggesting potential competition between these post-translational modifications with possible regulatory implications (Kolbert et al., 2017). Together, we identified numerous PTMs, most of which were previously unreported, likely due to low *in vivo* protein abundance. As the analysis was limited to mesophyll protoplasts under a single condition, additional PTMs are likely to occur in other cell types and stress contexts.

In this study we examined the potential relevance of selected PTMs in CM and PAT. Which catalyze the last two shared steps in Phe and Tyr biosynthesis (**Figure 1**). In particular, CM catalyzes the commited step into the Phe and Tyr pathway and importantly, its plastidial isoform is feedback regulated (El-Azaz and Maeda, 2025). As we have shown, using GFP-tagged proteins, we were unable to detect alterations in the subcellular localization of AtCM1 phosphomimic mutants Ser244Asp or Ser184Asp compared to wild type AtCM1. Oppositely, and very interestingly, the mutant protein AtCM2 Thr72Asp results in an altered accumulation of the protein in the cytosol compared to AtCM2. This alteration consists of the formation of puncta in the cytosol, which contrast with the uniform distribution of AtCM2-GFP wild type protein. The location of Thr72 on the dimerization surface of the enzyme suggests that phosphorylation of this residue could affect the interaction between monomers resulting in a distinctive distribution pattern that could have regulatory implications.

Since AtCM1, in contrast to AtCM2, is involved in Phe and Tyr biosynthesis within the plastids and undergoes allosteric regulation, we opted to explore the effect that phosphorylations of Ser244 and Ser184 may have on AtCM1 activity. Our results revealed that mimicking the phosphorylations of Ser184 or Ser244, by mutating to Asp, resulted in opposite effects on *k_cat_*, which increased by twofold in AtCM1^S244D^, but was reduced by half in AtCM1^S184D^, compared to wild-type AtCM1. These results suggest the existence of a finely tuned mechanism regulating AtCM1 activity via alternative phosphorylation events, which may be highly relevant, as they could regulate the rate of synthesis of prephenate and subsequently that of Phe and Tyr (**Supplementary Figure 4**). In this regard, it is important to note that CM competes with AS for the same substrate, chorismate (**Figure 1**), and thus an increase or decrease in the *k_cat_*in CM could have a significant effect on chorismate usage for Trp versus Phe/Tyr production (**Supplementary Figure 4**). We have also shown that the mimicry of the phosphoresidue at Ser244, results in an enzyme with enhanced inhibition by Phe and Tyr. This effect might be also of importance for modulating Phe/Tyr *vs*. Trp levels under changing metabolic conditions. This observation is consistent with the location of Ser244 within the 3D structure in the vicinity of residues described as critical for the allosteric regulation of the enzyme (Westfall et al., 2014) but also exposed to the surface of the protein, and thus accessible to kinases (**Figure 5A**).

We also evaluated the potential regulatory role of two phosphorylation events detected on PAT residues Ser240 and Ser247. *In vitro* activity assays confirmed that phosphomimetic substitutions at these two residues yield enzymes lacking PAT activity. The presence of Ser240 and Ser247 at highly conserved positions across plant PAT sequences strongly suggests that their regulatory role may be evolutionarily conserved. Structural analysis based on the available open-conformation crystal structure of Arabidopsis PAT (PDB: 6F5V; Giustini et al., 2019) indicates that both residues are located in an internal region adjacent to the protein surface and not directly solvent-exposed (**Figure 9**). However, phosphorylation of partially buried or solvent-inaccessible residues has been reported in the literature, indicating that limited solvent accessibility does not necessarily preclude regulatory phosphorylation (Jimenez et al., 2007). In addition, we cannot exclude the possibility that the accessibility of Ser240 and Ser247 differs in the closed conformation of the enzyme, although this cannot be evaluated at present because no structural model of the closed state is available.

Together, these findings support that PTMs of CM and PAT may have an important role to fine-tune metabolic flux into the post-chorismate pathways, suggesting that other AAA biosynthetic enzymes may also be subjected to similar mechanisms. Collectively, these results provide a valuable foundation for the identification of novel regulatory mechanisms with strong biotechnological potential to engineer AAA and natural products metabolism in plants.

## Materials and methods

### DNA constructs

Cloning of the coding region from AtADT1, AtADT2, AtADT3, AtADT4, AtADT5 and AtADT6 was previously described (El-Azaz et al., 2016, El-Azaz et al., 2018, El-Azaz et al., 2022). The remaining genes corresponding to the shikimate pathway and Phe synthesis pathway were cloned from cDNA libraries obtained from leaves, stems and flowers from five-week-old Arabidopsis ecotype Columbia (Col-0) plants grown under 14/10 hr light/dark regimen (150 μE·m^-2^·s^-1^) at 22°C. All cloning procedures were performed using iProof DNA Polymerase (Bio-Rad) and the oligonucleotides listed in **Supplementary Table 1**. PCR amplifications were carried out in a T100 Thermal Cycler (Biorad) using annealing temperatures ranging from 50 to 55 °C, depending on the primer pair employed. The expression of C-terminal GFP and FLAG fusion proteins in Arabidopsis protoplasts was conducted using gateway compatible vectors, pUCGWB5 and pUCGWB11, generated in our laboratory by respectively combining the HindIII/SacI fragments of pGWB5 and pGWB11 (courtesy of Dr Tsuyoshi Nakagawa, Department of Molecular and Functional Genomics, Shimane University, Japan) into the pUC19 vector (Yanisch-Perron et al., 1985). Briefly, the corresponding CDS regions were PCR amplified with oligonucleotides that included the attB1 and attB2 sequences (**Supplementary Table 1**), purified from agarose gels and cloned into Gateway^®^ pDONR207™ (Thermo Fisher Scientific) donor vector using BP Clonase^®^ II enzyme mix (Thermo Fisher Scientific). Subsequently, these clones were recombinated into the mentioned destination vectors using the Gateway^®^ LR Clonase^®^ II mix (Thermo Fisher Scientific).

### Site-directed mutagenesis

Site-directed mutagenesis was performed to generate AtCM1, AtCM2 and AtPAT phosphomimic mutant versions using iProof high-fidelity DNA polymerase (Bio-Rad). Briefly, 10 ng of the pUCGWB5-*AtCM1*, pUCGWB5-*AtCM2* and pUCGWB5-*AtPAT* vectors were used for PCR amplification using the corresponding oligonucleotides described in **Supplementary Table 1** under the following conditions: 2 min at 98 °C, followed by 23 cycles (10 s at 98 °C, 20 s at 55 °C and 4 min at 72 °C) and a final elongation of 4 min at 72 °C. Samples were treated with DpnI (ThermoScientific™) to eliminate native methylated plasmid DNA, and the PCR product was transformed into *Escherichia coli* DH5α with selection on 100 μg ml−1 ampicillin. Phosphomimetic mutant versions for the production of recombinant proteins in *E. coli* were generated using pDEST17-*AtCM1ΔcTP*, pDEST17-*AtCM2* and pDEST17-*AtPATΔcTP* vectors as templates and following identical procedure.

### Protoplast isolation and transfection

Protoplast transformation was performed according to the protocol described by Wu et al. (2009) with minor modifications. Briefly, protoplasts were prepared from fully expanded leaves from 4-weeks-old Arabidopsis plants grown in a 14/10 hr light/dark regimen (150 μE·m^-2^·s^-1^) at 22°C. The upper epidermal surface of 30/40 leaves were fixed to a 50 cm long strip of Time tape (Time Med, Burr Ridge, IL) while the lower epidermal surface was covered with Magic tape (3 M, St. Paul, MN). Following, the Magic tape was removed carefully removing the lower epidermal surface cell layer. The peeled leaves attached to the Time tape were arranged facing downwards in Petri dishes containing 12.5 mL of enzyme solution (1% cellulase from Duchefa, 0.25% macerozyme also from Duchefa, 0.4 M mannitol, 10 mM CaCl_2_, 20 mM KCl, 0.1% BSA and 20 mM MES, pH 5.7). Plates were gently shaken in light for 60/70 min until the solution became green due to the releasing of protoplasts. The suspension of protoplasts was centrifuged at 100xg for 4 min and 15°C in a Hettich Universal 320R centrifuge using a Hettich 1619 swing-out rotor with the brake disabled. The pellet of protoplasts was washed twice with 25 mL of cold W5 solution (154 mM NaCl, 125 mM CaCl_2_, 5 mM KCl, 5 mM glucose, and 2 mM MES, pH 5.7) and incubated on ice for 30 min. At this point, the protoplasts were quantified and their integrity was checked using an optical microscope and serial dilutions of the preparation. The suspension of protoplasts was subsequently centrifuged at 100xg for 1 min and the pellet resuspended in MMg solution (0.4 M mannitol, 15 mM MgCl_2_, and 4 mM MES, pH 5.7) to a final concentration of 1x10^6^ cells/mL. Then, 200 µL of the suspension of protoplast, containing around 2x10^5^ cells, were transferred to a round-bottom polypropylene tube and mixed with 50 µL of water containing 30 µg of plasmid DNA and 250 µL of the PEG solution (40% w/v of PEG-4000, 0.1 M CaCl_2_ and 0.2 M mannitol). After 15 min, 3 mL of cold W5 solution were added to each tube, mixed by gently inversion and protoplasts were collected by centrifugation at 100xg. This washing step was repeated twice. Protoplasts were resuspended in 1 mL of W5 and incubated in 6-well plates coated with 1% BSA at room temperature for 16/20 hr in light subsequently collected by centrifugation and stored at -80°C.

### Immunoprecipitation of proteins

Protoplast pellets were thawed and soluble proteins were extracted in lysis buffer [50mM Tris-HCl pH 7.5, 150 mM NaCl, 10% glycerol, 2 mM EDTA, 1% Triton X-100, 1% protease inhibitor cocktail (Sigma P9599) and 1% phosphatase inhibitor cocktail (PhosSTOP, Roche)]. Vigorous vortex shaking for 30 sec was applied in order to completely break up the protoplasts. The preparation was subjected to centrifugation for 20 min at 4°C and the supernatant collected and immunoprecipitation was initiated by the addition of agarose beads (Flag-tag, DYKDDDDK Fab-Trap, Chromotek-Proteintech) to the supernatant. After 1 h incubation at 4 °C and three washes with lysis buffer, proteins were eluted by incubating the beads with 2x Laemmli buffer at 100 °C for 5 minutes. After a brief spin, proteins were transferred to a new tube, frozen in liquid nitrogen, and stored at -80 °C.

### Identification of PTMs

Immnuprecipitated proteins were loaded onto 10% SDS-PAGE gels and allowed to migrate 1 cm into the gel, and briefly stained with Coomassie Blue R-250. The 1cm gel section was excised from the Coomassie-stained gel, destained, digested with trypsin, and subject to mass spectrometric analysis. Briefly, gel slices containing fusion proteins were reduced with 10 mM DTT, alkylated with 55 mM iodoacetamide, and then digested overnight at 37°C with sequencing grade trypsin (Sigma, USA). The tryptic peptides were subject to LC-MS/MS analysis using an Orbitrap Fusion mass spectrometer with enabled multistage activation. Proteome Discoverer software (version 2.5) (Thermo Fisher) was used for peptide identification and PTM assignment. The *Arabidopsis thaliana* proteome sequences (Uniprot) were used as the data-base and the mass tolerances were set to 10 ppm for precursor and 0.2 Da for fragment ions, and the *Arabidopsis thaliana* proteomedatabase (Uniprot) was searched. The mass spectrometry proteomics data have been deposited to the ProteomeXchange Consortium via the PRIDE (Perez-Riverol et al., 2025) partner repository with the dataset identifier PXD082490.

### Western blot

For the immunodetection of transiently expressed proteins in protoplasts, approximately 30 μg of total proteins were separated using sodium dodecyl sulfate polyacrylamide gel electrophoresis (SDS-PAGE). Western blot analysis was conducted following standard procedures. GFP and FLAG tagged proteins were detected by exploiting the corresponding C-terminal tags present in the constructs, utilizing specific commercial antibodies. In both cases mouse monoclonal antibodies from Santa Cruz Biotechnology (Santa Cruz, CA, USA) were used to detect fusion proteins: GFP (B-2) sc-9996 at a dilution of 1:1000 for GFP and OctA-Probe (H-5) sc-166355 at a dilution of 1:500 for FLAG.

### Recombinant expression and purification

DNA sequences coding for AtCM1, AtPAT and phosphomimic mutant versions were subcloned into the *pDEST17* vector using Gateway technology. The sequence coding for the putative chloroplast transit peptides of AtCM1 (residues 1–58), and AtPAT (residues 1-54), were not included. These sequences were determined by comparison with multiple sequences from plants, using ChloroP (Emanuelsson et al., 1999) and TargetP 2.0 algorithms (Almagro Armenteros et al., 2019). *pDEST17* expression constructs were transformed into *Escherichia coli* BL21-CodonPlus-RIL^®^ (Agilent Technologies, Santa Clara, CA, USA) and cultured in 700 mL of LB, supplemented with 100 µg of ampicillin and 34 µg of chloramphenicol, at 37 °C until A_600_ of 0.5/0.6 was reached. Protein expression was induced by adding a final concentration of 1 mM isopropyl β-D-1-thiogalactopyranoside (IPTG) to the cultures, which were subsequently incubated for 16 h at 10 °C with gentle shaking at 100 rpm. Cells were pelleted by centrifugation at 5000×g and frozen until further purification. Poly-His-tagged recombinant proteins were detected by western blot using the mouse monoclonal antibody His-Tag (H-3): sc-8036 from Santa Cruz Biotechnology (Santa Cruz, CA, USA). Purification was conducted using Protino Ni-TED 2000 Packed nickel resin columns (Macherey-Nagel, Duren, Germany) and subjected to buffer exchange to a 50 mM Tris buffer at pH 8.0 using Sephadex G-25 M resin (PD-10 Columns; GE Healthcare, Chicago, IL, USA).

### CM activity assay

CM reactions were conducted in a final volume of 80 µL containing 50 mM Tris buffer pH 8, varying concentrations of chorismate, and 10–100 ng of the corresponding purified CM enzyme. CM assays were conducted in a plate reader at 30 °C, using UV-transparent 96-well plates (UV-Star^®^, Greiner Bio-One. GmbH, Frickenhausen, Germany), by tracking the disappearance of chorismate, which results in an absorbance decrease at A_274_ nm (ϵ = 2630 M^−1^ cm^−1^) or A_310_ nm (ϵ = 370 M^−1^ cm^−1^). Chorismate (C1259) was obtained from Merck KGaA (Darmstadt, Germany). To identify putative amino acid effectors, the reactions were conducted as described above using 0.5 mM chorismate and supplemented with 2 mM of each aromatic amino acid: Phe, Tyr and Trp.

### PAT activity assay

PAT activity was determined by coupling the production of oxaloacetate, resulting from Asp transamination, to the oxidation of NADH using malate dehydrogenase. Reactions were developed in a mix solution containing 50 mm Tris-HCl, pH 8, 20 mm Asp, 1 mm prephenate, 200 µM PLP, 0.1 mm NADH, 2 units of malate dehydrogenase (Roche Farma), and 300 ng of the recombinant PAT enzymes in a final volume of 200 *μ*L. The reaction was started by the addition of the PAT enzyme and the decrease of NADH levels was measured at 340 nm for a period of 600 s. PAT assays were conducted in a plate reader at 30 °C, using UV-transparent 96-well plates (UV-Star^®^, Greiner Bio-One. GmbH, Frickenhausen, Germany).

### Subcellular localization

AtCM1, AtCM2, AtPAT and mutant versions were cloned into the pUCGWB5 vector using gateway technology to create complete CM or PAT proteins tagged with GFP at their C-termini, under the regulation of the CaMV 35S promoter. The resulting plasmids were transformed into Arabidopsis protoplasts using polyethylene glycol (PEG)‐mediated DNA transfection (Yoo et al., 2007; Wu et al., 2009). GFP fluorescence was assessed 20 h post-transformation using a Leica Stellaris 8 confocal microscope (Leica, Wetzlar, Germany), for chloroplast autofluorescence of excitation/emission at 488/680–700 nm and for GFP detection excitation/emission at 488/505–525 nm. Excitation was provided by an argon ion laser 488 nm.

## Data Availability

The original contributions presented in the study are included in the article/**Supplementary Material**. Further inquiries can be directed to the corresponding authors.

## Supplementary material

Supplementary Figure 1 Multiple sequence alignment of plant CMs.

Supplementary Figure 2 *In vitro* kinetic characterization of recombinant AtCM1 and phosphomimetic versions.

Supplementary Figure 3 Confocal GFP imaging of AtPAT and phosphomimetic variants in Arabidopsis protoplasts.

Supplementary Figure 4 Model of AtCM1 kinetic regulation via phosphorylation.

Supplementary Table 1 Oligonucleotides used in this work.
