# Supplementary material for: Complex post-translational regulation of the plant shikimate pathway
Source: Front Plant Sci. 2026 Oct 9;17:1848534. doi: 10.3389/fpls.2026.1848534 (PMC13690941; doi:10.3389/fpls.2026.1848534)
Supplement: Supplementary file 1 [file Table1.docx]

**Supplementary Table 1. Oligonucleotides used in this work.**

| **Oligo name** | **Oligo purpose** | **Sequence** |
| --- | --- | --- |
| Phe041 | AtDAHPS1 AT4G39980 CDS Gateway cloning Forward attb1 | AAAAAGCAGGCTTAATGGCTCTTTCCAACGCCTCC |
| Phe042 | AtDAHPS1 AT4G39980 CDS Gateway cloning Reverse attb2 | AGAAAGCTGGGTTAGACACACGCTGGCTACCCG |
| Phe043 | AtDAHPS2 AT4G33510 CDS Gateway cloning Forward attb1 | AAAAAGCAGGCTTAATGGTGACTCTAAACGCTTCTTCTCC |
| Phe044 | AtDAHPS2 AT4G33510 CDS Gateway cloning Reverse attb2 | AGAAAGCTGGGTTGACTCCAATAGATGACGGAAG |
| Phe045 | AtDAHPS3 AT1G22410 CDS Gateway cloning Forward attb1 | AAAAAGCAGGCTTAATGGCGCTTATGAATGGAAGC |
| Phe046 | AtDAHPS3 AT1G22410 CDS Gateway cloning Reverse attb2 | AGAAAGCTGGGTTGAGGGCAAAAGCCTTTTGAGACTTG |
| Phe003 | AtDHQS AT5g66120 CDS Gateway cloning Forward attb1 | AAAAAGCAGGCTTAATGGCAGCCAACACCATTTCTCTTTCC |
| Phe004 | AtDHQS AT5g66120 CDS Gateway cloning Reverse attb2 | AGAAAGCTGGGTTGGATTTGGAGAATGCACGAAGCG |
| Phe007 | AtDQD/SDH AT3G06350 CDS Gateway cloning Forward attb1 | AAAAAGCAGGCTTAATGGCAGCTTCTTCAACTAATGC |
| Phe008 | AtDQD/SDH AT3G06350 CDS Gateway cloning Reverse attb2 | AGAAAGCTGGGTTGTACTTTGACATTATTTGCCAG |
| Phe011 | AtSK1 At2G21940 CDS Gateway cloning Forward attb1 | AAAAAGCAGGCTTAATGGAAGCAGCTATTACTC |
| Phe012 | AtSK1 At2G21940 CDS Gateway cloning Reverse attb2 | AGAAAGCTGGGTTGAGGTCGCCGTCTGGGATCTCC |
| Phe053 | AtSK2 AT4G39540 CDS Gateway cloning Forward attb1 | AAAAAGCAGGCTTAATGGAAGCAGCTACTGTTCAGAGG |
| Phe054 | AtSK2 AT4G39540 CDS Gateway cloning Reverse attb2 | AGAAAGCTGGGTTGAGTCCATCTGGTCTAGCC |
| Phe055 | AtEPSPS1 AT2G45300 CDS Gateway cloning Forward attb1 | AAAAAGCAGGCTTAATGGCGCAAGTTAGCAGAATCTGC |
| Phe056 | AtEPSPS1 AT2G45300 CDS Gateway cloning Reverse attb2 | AGAAAGCTGGGTTGTGCTTTGTGATTCTTTCAAGTACTTGG |
| Phe057 | AtEPSPS2 AT1G48860 CDS Gateway cloning Forward attb1 | AAAAAGCAGGCTTAATGGCGTCTTCTCTCACTTCC |
| Phe058 | AtEPSPS2 AT1G48860 CDS Gateway cloning Reverse attb2 | AGAAAGCTGGGTTATGCTTTGTGATTCTTTCAAGGAC |
| Phe059 | AtCS AT1G48850 CDS Gateway cloning Forward attb1 | AAAAAGCAGGCTTAATGGCGTCGTCTTCTCTC |
| Phe060 | AtCS AT1G48850 CDS Gateway cloning Reverse attb2 | AGAAAGCTGGGTTCAAAGCAGTAGCATTTTGCGG |
| Phe047 | AtCM1 AT3G29200 CDS Gateway cloning Forward attb1 | AAAAAGCAGGCTTAATGGAAGCGTCATTGTTGATGAG |
| Phe048 | AtCM1 AT3G29200 CDS Gateway cloning Reverse attb2 | AGAAAGCTGGGTTGTCCAGTCTTCTGAGCAAG |
| Phe049 | AtCM2 AT5G10870 CDS Gateway cloning Forward attb1 | AAAAAGCAGGCTTAATGGCAAGAGTCTTCGAATCGG |
| Phe050 | AtCM2 AT5G10870 CDS Gateway cloning Reverse attb2 | AGAAAGCTGGGTTATCGAGACGACGTAGAAGATAC |
| Phe051 | AtCM3 AT1G69370 CDS Gateway cloning Forward attb1 | AAAAAGCAGGCTTAATGGAGGCTAAGTTACTCAAACC |
| Phe052 | AtCM3 AT1G69370 CDS Gateway cloning Reverse attb2 | AGAAAGCTGGGTTATCCAGTCTTCTAAGCAAG |
| Phe065 | AtPAT AT2G22250 CDS Gateway cloning Forward attb1 | AAAAAGCAGGCTTAATGGCTTCTCAGAGTTCAG |
| Phe070 | AtPAT AT2G22250 CDS Gateway cloning Reverse attb2 | AGAAAGCTGGGTTAACGGAGACAGTGGCACGG |
| Phe061 | AtADT1 AT1G11790 CDS Gateway cloning Forward attb1 | AAAAAGCAGGCTTAATGGCTCTGAGGTGTT |
| AtADT1-attB2 | AtADT1 AT1G11790 CDS Gateway cloning Reverse attb2 | AGAAAGCTGGGTTGTTATCTGACTAGATCC |
| AtADT2-attB1 | AtADT2 AT3G07630 CDS Gateway cloning Forward attb1 | AAAAAGCAGGCTTAATGGCAATGCACACTGTTCG |
| AtADT2-attB2 | AtADT2 AT3G07630 CDS Gateway cloning Reverse attb2 | AGAAAGCTGGGTTGAGCATTGTAGTGTCCACTGG |
| AtADT3-attB1 | AtADT3 AT2G27820 CDS Gateway cloning Forward attb1 | AAAAAGCAGGCTTAATGAGAACTCTCTTACCTTCC |
| AtADT3-attB2 | AtADT3 AT2G27820 CDS Gateway cloning Reverse attb2 | AGAAAGCTGGGTTCAATGAAAATGTTGATGACG |
| Phe062 | AtADT4 AT3G44720 CDS Gateway cloning Forward attb1 | AAAAAGCAGGCTTAATGCAAGCCGCAACGTCG |
| AtADT4-attb2 | AtADT4 AT3G44720 CDS Gateway cloning Reverse attb2 | AGAAAGCTGGGTCTGCTTCTTCTGTGGATGTCATGG |
| Phe063 | AtADT5 AT5G22630 CDS Gateway cloning Forward attb1 | AAAAAGCAGGCTTAATGCAAACCATTTCGCCTGC |
| AtADT5-attB2 | AtADT5 AT5G22630 CDS Gateway cloning Reverse attb2 | AGAAAGCTGGGTTTACGTCTTCGCTAGGTAACG |
| Phe064 | AtADT6 AT1G08250 CDS Gateway cloning Forward attb1 | AAAAAGCAGGCTTAATGAAAGCTCTATCATC |
| Phe069 | AtADT6 AT1G08250 CDS Gateway cloning Reverse attb2 | AGAAAGCTGGGTTCGATGAAGTTGATGATGTTGG |
| Phe244 | Phosphomimic mutation of AtCM1 Ser184Asp Forward | GCATTTTGCTGCTGATGACATAAACATAAACAAG |
| Phe245 | Phosphomimic mutation of AtCM1 Ser184Asp Reverse | CTTGTTTATGTTTATGTCATCAGCAGCAAAATGC |
| Phe248 | Phosphomimic mutation of AtCM1 Ser244Asp Forward | GCTAAATTTCAAGCCGACCCCGAAGCATACG |
| Phe249 | Phosphomimic mutation of AtCM1 Ser244Asp Reverse | CGTATGCTTCGGGGTCGGCTTGAAATTTAGC |
| Phe265 | Phosphomimic mutation of AtCM2 Thr72Asp Forward | CGTCAGAGAGGATGAAATCATCCAAGCT |
| Phe266 | Phosphomimic mutation of AtCM2 Thr72Asp Reverse | AGCTTGGATGATTTCATCCTCTCTGACG |
| Phe267 | Phosphomimic mutation of AtCM2 Thr223Asp Forward | GGACAAGAAGTAAAATTCAACGATGGCTATGGCG |
| Phe268 | Phosphomimic mutation of AtCM2 Thr223Asp Reverse | CGCCATAGCCATCGTTGAATTTTACTTCTTGTCC |
| ctpCM1 attb1 | AtCM1 AT3G29200 CDS minus cTP Gateway Forward attb1 | AAAAAGCAGGCTTAATGTCTGGAACCAGCTCTGTTCACG |
| AtCM1 Stop RV | AtCM1 AT3G29200 CDS Gateway cloning Reverse STOP attb2 | AGAAAGCTGGGTTCAGTCCAGTCTTCTGAGC |
| Phe264 | AtCM2 AT5G10870 CDS Gateway cloning Reverse STOP attb2 | AGAAAGCTGGGTTCAATCGAGACGACGTAGAAGATAC |
| Phe319 | Phosphomimic mutation of AtPAT Ser240Asp Forward | GACTTCTTATTCTCTGCGATCCTTCCAACCCTACTGG |
| Phe320 | Phosphomimic mutation of AtPAT Ser240Asp Reverse | CCAGTAGGGTTGGAAGGATCGCAGAGAATAAGAAGTC |
| Phe167 | Phosphomimic mutation of AtPAT Ser247Asp Forward | CCAACCCTACTGGAGATGTTTACCCCAAGAG |
| Phe168 | Phosphomimic mutation of AtPAT Ser247Asp Reverse | CTCTTGGGGTAAACATCTCCAGTAGGGTTGG |
| Phe169 | AtPAT Gateway cloning Forward attb1 minus CTP | AAAAAGCAGGCTTAGCTGAGACTCTTTCATCATCAGTAG |
